# Static-state particle fabrication via rapid vitrification of a thixotropic medium

**DOI:** 10.1038/s41467-021-23992-2

**Published:** 2021-06-18

**Authors:** Sang Yup Kim, Shanliangzi Liu, Sungwoo Sohn, Jane Jacobs, Mark D. Shattuck, Corey S. O’Hern, Jan Schroers, Michael Loewenberg, Rebecca Kramer-Bottiglio

**Affiliations:** 1 Department of Mechanical Engineering and Materials Science, Yale University, New Haven, CT USA; 2 Department of Mechanical Engineering, Sogang University, Seoul, Republic of Korea; 3 School of Mechanical Engineering, Purdue University, West Lafayette, IN USA; 4 Department of Physics, City University of New York, New York, NY USA; 5 Department of Chemical and Environmental Engineering, Yale University, New Haven, CT USA

**Keywords:** Colloids, Composites, Design, synthesis and processing

## Abstract

Functional particles that respond to external stimuli are spurring technological evolution across various disciplines. While large-scale production of functional particles is needed for their use in real-life applications, precise control over particle shapes and directional properties has remained elusive for high-throughput processes. We developed a high-throughput emulsion-based process that exploits rapid vitrification of a thixotropic medium to manufacture diverse functional particles in large quantities. The vitrified medium renders stationary emulsion droplets that preserve their shape and size during solidification, and energetic fields can be applied to build programmed anisotropy into the particles. We showcase mass-production of several functional particles, including low-melting point metallic particles, self-propelling Janus particles, and unidirectionally-magnetized robotic particles, via this static-state particle fabrication process.

## Abstract (web summary)

Upscale fabrication of functionalized microparticles is a pending challenge. Here, Kim et al. exploit the rheology of a thixotropic medium to grind sizeable amounts of raw material into well-defined colloidal dispersions, physically stabilized for further production steps.

## Introduction

Particles are extensively used in various disciplines, such as medicine^1–3^, materials science^4–6^, and robotics^7,8^. Such minute fragments of matter are often incorporated into bulk materials to achieve multifunctional composites^9–11^, dispersion inks^12,13^, and granular metamaterials^14^, which yield gained functionalities responsive to external stimuli. Thus, mass manufacturing of functional particles is becoming increasingly important to translate particulate material discoveries to large-scale applications. One widespread method to mass-produce particles from bulk matter is emulsion processing^15–17^ that consists of emulsifying a bulk liquid in a continuous phase via either shear mixing or sonication, then solidifying the distributed phase. Although rapid and scalable, previous emulsion processes have not addressed the need for high process yield^18^, monodispersity^19,20^, or anisotropy^21,22^. Similarly, micro-fluidic particle fabrication processes can address these critical demands, but produce small numbers of particles (large quantity production is time-consuming and costly).

Throughout an emulsion process, sedimentation or creaming of the distributed phase (emulsion droplets) after their creation and prior to their solidification result in droplet coalescence, causing irregular clumps of particles and potentially ruining the overall particle batch^23,24^. This phenomenon is usually prevented by stirring the continuous phase until the distributed droplets are fully solidified^25–27^, but this redundant shearing causes the droplets to collide and deform, ultimately leading to low process yield and high particle heterogeneity. One proposed solution to this challenge is to fix and isolate each droplet in place by vitrifying the continuous phase after emulsification. This static-state particle fabrication process can eliminate coalescence and droplet deformation, but existing methods require secondary steps to vitrify the continuous phase (*e.g*. freezing^28–30^, adding thickening agent^31,32^) which are too complex and laborious to scale production volume.

Here, we report a method of fabricating particles via a scalable, static-state emulsion process. The method uses a shear-thinning thixotropic medium as the continuous phase that reduces its viscosity with increasing shear and then rapidly vitrifies when the shearing stops. This rheological response of the continuous phase enables both dynamic-state emulsification (during shear mixing) and static-state solidification (with the mixer disengaged), all within the same batch and without additional processing steps. Moreover, once the particles are held in place in the continuous phase, energetic fields can penetrate the continuous phase to induce anisotropy in the solidifying particles, enabling scalable programming of the intrinsic particle properties. Our method herein is highly generalizable as long as the continuous phase exhibits thixotropy, and in this study, gelatinized corn starch in water is used as the medium, which carries the additional advantage of being eco-friendly with sustainable by-products.

## Results and discussion

### Overview of the static-state particle fabrication

**Fig. 1:**
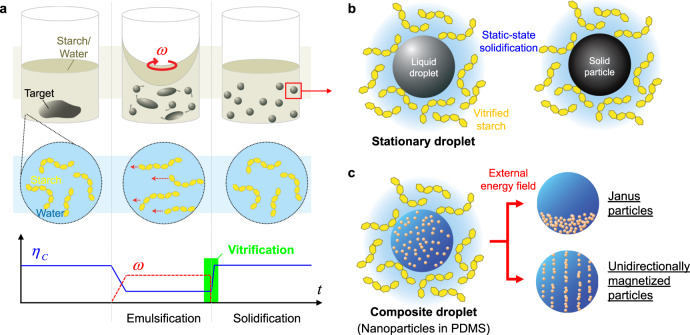
Overview of the static-state particle fabrication process. **a** The viscosity of the starch/water medium ${\eta }_{C}$ decreases with the presence of a shear force (shear rate *ω*) in the emulsification stage, and abruptly increases to the initial value when the shearing is stopped. This abrupt increase in ${\eta }_{{\rm{C}}}$ gives rise to the vitrification of the medium, fixing the distributed phase (emulsion droplets) in place. **b** Stationary droplets isolated in the vitrified medium undergo the static-state solidification devoid of deformation forces or coalescence, enabling the droplets to preserve their shape and size during the solidification. **c** Scalable programming of anisotropy in particles is realized by emulsifying a mixture of nanoparticles and a PDMS resin to composite droplets. The nanoparticle inclusions are arranged by an external energy field inside the stationary polymer droplets, followed by curing of the polymer resin.

 Figure 1 shows the overview of our static-state particle fabrication process that enables scalable manufacturing and programming of functional particles. The target material is placed in bulk in the corn starch/water medium and broken up by shearing (Fig. 1a). As the shear rate *ω* increases, the viscosity *η*_*c*_ of the continuous phase drops and emulsifies the target material. The shearing is stopped after the emulsification and *η*_*c*_ increases by ~100 times to rapidly vitrify the continuous phase. The emulsion droplets are seized by networks of starch within the vitrified medium and remain suspended without creaming, sedimentation, or colliding with each other during the subsequent solidification (Fig. 1b). This static-state condition unlocks scalable programming of anisotropy in particles when a mixture of nanoparticles and a polydimethylsiloxane (PDMS) resin is utilized as the target material (Fig. 1c). The arrangement of nanoparticles, inside stationary resin droplets, is controlled by an external energetic field to realize anisotropic particles. In addition, prepared particles are easily retrieved by adding an enzyme to catalyze the hydrolysis of starch.

### Scalable manufacturing of low-melting point alloy particles

**Fig. 2:**
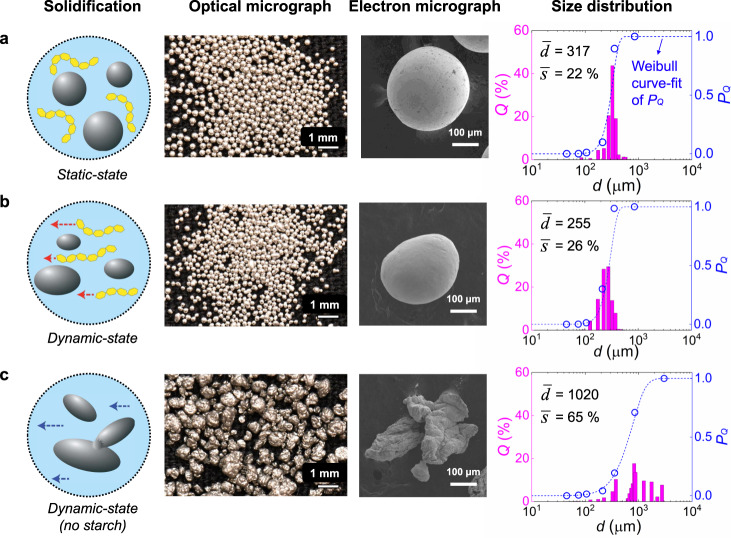
Field’s Metal particles fabricated under different conditions. **a** Static-state process in starch/water medium is devoid of any external force or internal particle coalescence during the solidification stage. Produced solid particles preserve the spherical shape of the emulsified droplets, and the particle size distribution is narrow with the mean particle diameter $\bar{d}$ = 317 μm with the standard deviation $\bar{s}$ = 22%. The particle size distribution is shown in the volume fraction, *Q* achieved from the image analysis, or in the cumulative mass, ${P}_{Q}$ achieved from the sieve analysis. **b** Dynamic-state process in starch/water medium mitigates internal particle coalescence but the continued shear force results in deformed particle shapes. **c** Dynamic-state process in water medium gives rise to both particle deformation and coalescence, resulting in particles with irregular shapes and a wide size distribution of $\bar{s}$ = 65%.

We first demonstrate our method using Field’s metal (FM), a Bi–In–Sn alloy with a low melting temperature of 62 °C, as the distributed phase, as shown in Fig. 2. A bulk of molten FM is emulsified in a medium at 90 °C and solidified as the medium cools below the melting temperature of the FM (see Methods for details). Previously, FM has been a challenging material to emulsify due to its high specific gravity (ca. 7.9), which causes rapid sedimentation before the droplets of molten FM cool and freeze^33^. However, using our method, the weight of the FM droplets is supported by the vitrified medium, allowing the particles to stay suspended during solidification (see Supplementary Movie 1). Utilizing a thixotropic medium also substantially enhances the quality of the produced FM particles. FM particles fabricated from our static-state fabrication process show a high degree of sphericity and surface smoothness (Fig. 2a). This morphology provides evidence that, in the absence of shear forces, the surface tension of the emulsion droplets dominates during solidification. Particle size analysis reveals a unimodal distribution with a standard deviation ca. 19%, and a process yield (the ratio of the total mass of retrieved particles to that of the input material) of >95%. Such a narrow size distribution and a high process yield are marked improvements compared to conventional emulsion-based processes, of which the process yield ranges from 50 to 80%^34,35^.

The underlying mechanism behind our static-state method is examined by comparing particles produced using the static- and dynamic-state methods. FM particles are deformed into ellipsoids when they are solidified within a viscous starch/water medium that is under continued shearing (Fig. 2b). Such particle morphology indicates that our static-state method eliminates the capillary stress applied to the emulsion droplets during their solidification, thereby preserving the spherical particle shape. When water alone is used as the continuous phase, irregularities in particle shape and size are further intensified (Fig. 2c). FM particles fabricated within a low-viscosity Newtonian medium (water) have a variety of shapes (*e.g*. ellipsoid, platelet, spiky sphere), and their sizes have a large standard deviation. We hypothesize that such heterogeneous particle shapes and sizes originate from high energy collisions of molten and solidified FM droplets, which is effectively mitigated when starch is present in the medium. The weakly perturbed environment of our static-state process also alters the microstructure of FM, enabling precise control of the particle quality (Supplementary Fig. 1 and Supplementary Note 1).

### Vitrification of a thixotropic medium

**Fig. 3:**
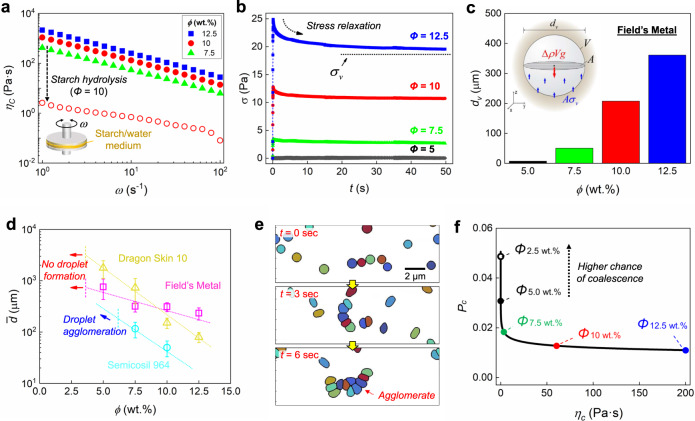
Characterization of the static-state particle fabrication process. **a** Rheological behavior of the starch/water medium. Either increasing a starch concentration *ϕ* in the medium or decreasing a shearing rate *ω* increases the viscosity of the medium ${\eta }_{{\rm{C}}}$. Hydrolysis of the starch reduces the viscosity ${\eta }_{{\rm{C}}}$, thereby facilitating the particle cleaning procedure. **b** Stress relaxation of the starch/water medium. The vitrification stress *σ*_v_ is defined where the residual stress plateaus after 1 h. **c** Vitrification diameter ${d}_{{\rm{v}}}$ of Field’s Metal (FM) particles in the starch/water medium. Increasing *ϕ* results in a higher ${d}_{{\rm{v}}}$ attributed to the increased ${\sigma }_{{\rm{v}}}$. **d** A relation of the concentration *ϕ* and the mean particle diameter $\bar{d}$ for polymer resins and FM. Two types of polymer resins are used: high-viscosity Dragon Skin 10 and low-viscosity Semicosil 964. Error bars denote one standard deviation. **e** Time-lapse images of simulated particle collision and coalescence behavior. Each particle is differently color-coded for clarity. **f** Coalescence probability *P*_c_ as a function of the viscosity ${\eta }_{{\rm{C}}}$. Starch concentration *ϕ* in correspondence to ${\eta }_{{\rm{C}}}$ reveals that a higher starch concentration in the medium mitigates the coalescence of the distributed phase.

We further investigate the underlying mechanism to optimize the processing parameters which will enable mass manufacturing of complex particles (Fig. 3). We draw specific data from the starch/water medium case previously described, but the principles can be expanded to the generalized system. The viscosity *η*_*c*_ of the continuous phase is controlled by the weight fraction *ϕ* of starch in the medium and the shearing rate *ω* (Fig. 3a). The increase of *η*_*c*_ in response to *ω* is nearly 2 orders of magnitude, which enables immediate vitrification once the shearing is stopped. In addition, higher *ϕ* increases *η*_*c*_ due to the higher density of gelatinized starch in the medium, and correspondingly, depolymerization of the starch (hydrolysis) decreases *η*_*c*_. Therefore, to retrieve prepared particles, we utilize hydrolysis of starch by adding enzyme, thereby upholding the eco-friendly and biocompatible process initially granted from using starch.

However, distributed droplets are still prone to sediment within the vitrified medium unless their weight can be supported by the viscoelastic stress of the vitrified medium. To measure the maximum value of the viscoelastic stress, the stress relaxation of the starch/water medium is examined (Fig. 3b). The stress applied to the medium is gradually reduced until it reaches a plateau as a function of time, and the value of the plateau point increases with increasing starch concentration *ϕ*. We use this plateau as the maximum value of the viscoelastic stress because it indicates that the medium remains stationary as long an external stress is less than the plateau value. We define this plateau value as the vitrification stress *σ*_v_ (see Methods for details), and since *σ*_v_ monotonically increases with *ϕ*, the medium can thus be tuned to support heavier droplets.

### Static-state condition of distributed droplets

The vitrification stress *σ*_v_, the density of the emulsion droplets ${\rho }_{{\rm{droplet}}}$, and the density of the medium ${\rho }_{{\rm{medium}}}$ allow a prediction of the maximum droplet size that will remain suspended within the medium as,

1 $$\varDelta \rho Vg\, <\, A{\sigma }_{v}$$

where $\varDelta \rho ={\rho }_{{\rm{droplet}}}-{\rho }_{{\rm{medium}}}$, *V* is the droplet volume, *g* is the gravitational acceleration, and *A* is the droplet cross-sectional area. Equation (1) can be simplified as $d\, <\, 1.5{\sigma }_{{\rm{v}}}\varDelta {\rho }^{-1}{g}^{-1}$ using the droplet diameter *d*, and therefore, droplets that do not exceed this specific size (hereby defined as the vitrification diameter, *d*_v_) can be indefinitely suspended within the vitrified medium. Figure 3c shows *d*_v_ of FM droplets (${\rho }_{{\rm{droplet}}}$ = 7,880 kg/m^3^) distributed within the starch/water medium, which reveals the ability of the vitrified medium to hold the heavy metallic droplets in place without sedimentation. For example, *d*_v_ of the FM droplets is ~200 μm at *ϕ* = 10 wt%, and only ~ 0.1 μm at *ϕ* = 5 wt%.

We examine how to control the diameter of emulsified droplets, such that the mean droplet diameter $\bar{d}$ is smaller than *d*_v_. The droplet diameter can be controlled during the emulsification primarily by tuning either the viscosity of the continuous phase *η*_C_ or the shearing rate *ω*. Figure 3d reveals that $\bar{d}$ decreases with increasing starch concentration *ϕ* under a fixed *ω*, because higher *ϕ* leads to higher *η*_C_. Therefore, higher *ϕ* is generally advantageous for enabling vitrification because it decreases $\bar{d}$ while simultaneously increasing *d*_v_ (see Fig. 3c). For example, under a given fixed *ω*, the FM droplets produced at *ϕ* = 7.5% are too large for the vitrified medium to support their weight ($\bar{d}$ = 317 μm vs *d*_v_ = 50 μm), but increasing to *ϕ* = 12.5% allows for droplet suspension in the vitrified medium ($\bar{d}$ = 230 μm vs *d*_v_ = 360 μm). In the case of a fixed *ϕ*, increasing *ω* can reduce $\bar{d}$ (Supplementary Fig. 2). Hence, both *ϕ* and *ω* can be controlled to satisfy the critical process condition of $\bar{d}$ < *d*_v_.

### Extension to polymeric distributed phase

To expand our method to polymeric particles, two types of polymer resins with different viscosities ${\eta }_{{\rm{D}}}$ are used as the distributed phase: Semicosil 964 (SC, ${\eta }_{{\rm{D}}}$ = 0.7 Pa s) and Dragon Skin 10 (DS, ${\eta }_{{\rm{D}}}$ = 23 Pa s). Compared to FM particles, polymeric particles are lower density (${\rho }_{{\rm{droplet}}}$~ 1,000 kg/m^3^), enabling *d*_v_ to reach tens of millimeters while maintaining compatibility with the vitrification process. For the emulsification, particle size analysis shows that SC particles are significantly smaller than DS particles under identical emulsification conditions (see Fig. 3d). This is presumably due to the difference in surface tension *γ* (Supplementary Fig. 3), as capillary stress ${\eta }_{{\rm{C}}}\omega$ applied to the distributed phase can emulsify the distributed phase as long as ${\eta }_{{\rm{C}}}\omega\, > \, \gamma {d}^{-1}$ is satisfied^36,37^. For the same reason, both DS and FM particles are unable to be fabricated at *ϕ* < 5 wt%, in contrast to SC particles which are successfully fabricated but then agglomerate (Supplementary Fig. 4).

### Mitigation of droplet agglomeration under shearing

Such agglomeration of SC particles at low *ϕ* suggests that the presence of starch mitigates particle coalescence, which is also demonstrated from the FM particles in Fig. 2c. To understand the particle coalescence process, we perform discrete element method simulations to model collisions of dispersed droplets in a shear field and calculate the coalescence frequency $\Omega$ = $C\times {P}_{{\rm{C}}}$, where *C* is the collision frequency and *P*_c_ is the coalescence probability^24^. Figure 3e shows a time-lapse simulation of individual particles moving in a shear field and agglomerating when gelatinized starch is absent (no inter-particle film drainage), due to particle collisions (Supplementary Movie 2). However, increasing ${\eta }_{{\rm{C}}}$ appears to have only a marginal effect on the collision frequency *C* (Supplementary Fig. 5) because *C* is mostly a function of particle density and shear rate alone^38^.

Therefore, the coalescence frequency $\Omega$ is primarily dependent on the coalescence probability ${P}_{{\rm{C}}}$, which is in turn a function of the viscosity of the medium ${\eta }_{{\rm{C}}}$. Not all particle collisions lead to coalescence; the form of the coalescence probability ${P}_{{\rm{C}}}$ can be described by

2 $${P}_{{\rm{C}}}={e}^{-{t}_{{\rm{c}}}/{t}_{{\rm{i}}}}$$

where ${t}_{{\rm{i}}}$ ~ ${\omega }^{-1}$ is the duration of a collision event, ${t}_{{\rm{c}}}$ ~ ${d}^{2}{\eta }_{{\rm{C}}}{f}^{-1}$ is the time required for droplets to coalesce, and $f$ is the interaction force between particles^39^. The relation between ${P}_{{\rm{C}}}$ and ${\eta }_{{\rm{C}}}$ in Fig. 3f reveals that ${P}_{{\rm{C}}}$ sharply increases with decreasing ${\eta }_{{\rm{C}}}$ when the initial ${\eta }_{{\rm{C}}}$ < 10 Pa s, which is consistent with the experimental results in Fig. 3d that show the departure of $\bar{d}$ of SC particles at *ϕ* < 5.0 wt%. These results indicate that the benefit of exploiting a thixotropic medium occurs during both the emulsification and solidification stages, and that our fabrication method is highly generalizable to various types of thixotropic media (*e.g*. hydrogel, syrup).

### Scalable programming of anisotropic particles

**Fig. 4:**
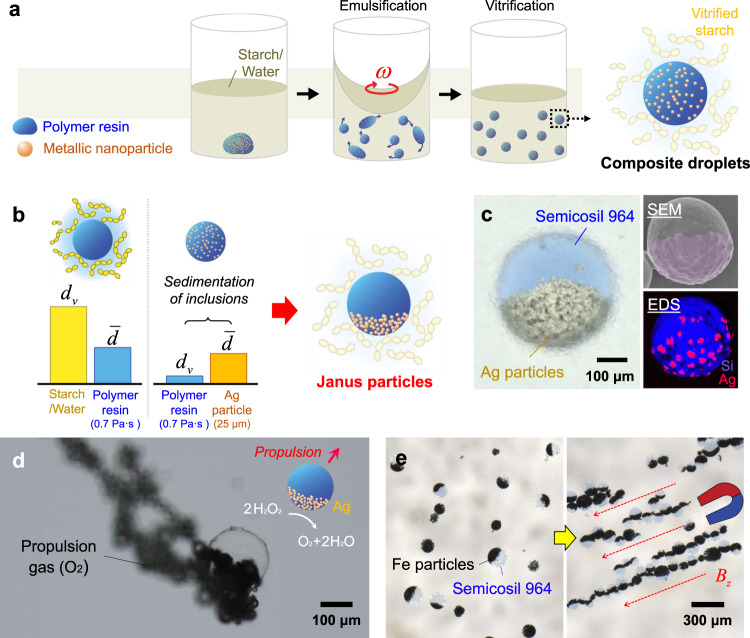
Scalable programming of Janus particles. **a** Illustration of preparing composite droplets to program anisotropy. **b** Manufacturing conditions for the composite droplets to evolve as Janus particles, determined by a relation between the vitrification diameter ${d}_{{\rm{v}}}$ and the mean diameter $\bar{d}$ of the droplet. Polymer droplets are fixed in a vitrified starch network (${d}_{v}$ > $\bar{d}$) while the nanoparticle inclusions sediment (${d}_{{\rm{v}}}$ < $\bar{d}$). **c** Optical micrograph of a Janus particle and its SEM and EDS images. The SEM image is false-colored to emphasize the presence of Ag on one side. **d** Self-propelling of the Janus particle in hydrogen peroxide. **e** Alignment of ferromagnetic Janus particles by an external magnetic field, *B*_z_.

To showcase the far-reaching impacts of our static-state particle fabrication method, we demonstrate the scalable programming of anisotropy in particles, as shown in Fig. 4. Anisotropic particles have promising applications in functional and intelligent materials, but their fabrication processes have thus far been unfeasible to scale-up^40,41^. Our static-state method addresses these challenges of mass-production by exploiting the relative difference in the ${d}_{{\rm{v}}}$ of metallic particles in a polymer resin and polymeric particles in a thixotropic medium. By emulsifying a mixture of metallic nanoparticles and uncured polymer resin, the metallic inclusions can be arranged inside larger polymer droplets using a variety of external forces before the polymer cures (Fig. 4a). The vitrified medium fixes the geometry and location of the composite droplets during this process, ensuring that each anisotropic particle maintains its size and shape as the internal inclusions flow inside the uncured resin.

We demonstrate two types of anisotropic particles—Janus particles and unidirectionally magnetized particles. Janus particles are made by leveraging the difference in densities between metal and polymer resin (${\rho }_{{\rm{metal}}} > > {\rho }_{{\rm{resin}}}$), which causes sedimentation of metallic inclusions ($\bar{d}$ > ${d}_{{\rm{v}}}$) within uncured polymer droplets due to gravity (Fig. 4b and Supplementary Movie 3). Figure 4c shows Janus particles composed of small Ag inclusions (${\rho }_{{\rm{inclusion}}}$= 10,490 kg/m^3^) arranged on one side of a larger PDMS droplet (${\rho }_{{\rm{droplet}}}$~ 1,000 kg/m^3^). These Janus particles can be used as self-propelling particles when they are placed in hydrogen peroxide, because the Ag catalyzes a process that generates oxygen bubbles (Fig. 4d and Supplementary Movie 4). In addition, when Ag inclusions are replaced with ferromagnetic Fe inclusions, prepared Janus particles can elicit a controlled group assembly under an external magnetic field, *B*_z_ (Fig. 4e). Similarly, mixtures of active and Brownian particles are predicted to generate unexpected topological defects and spacings, which could lead to dynamic self-assembly of crystalline and metamaterial structures^42^.

**Fig. 5:**
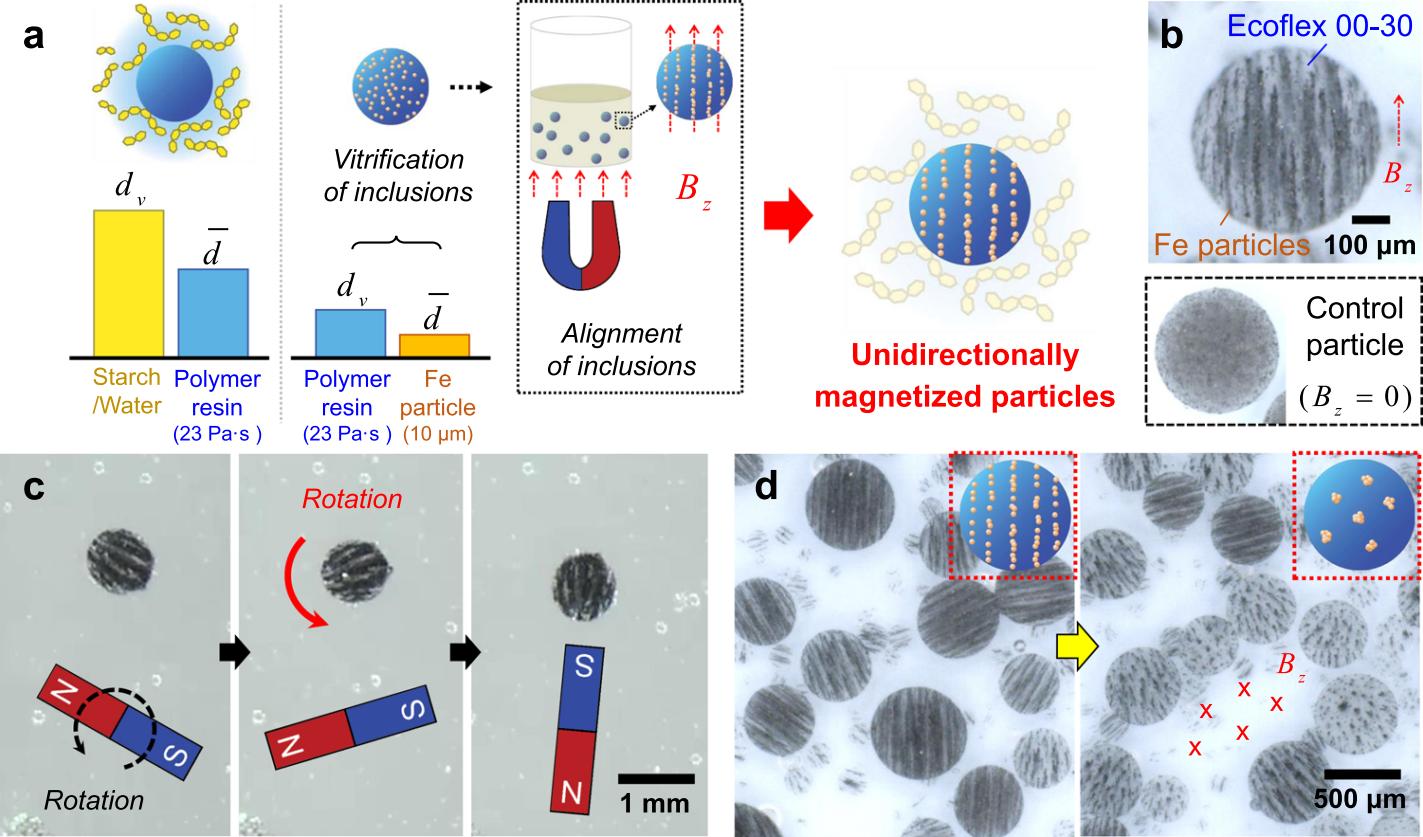
Scalable programming of unidirectionally magnetized particles. **a** Manufacturing conditions for the composite droplets to evolve as unidirectionally magnetized particles. An external magnetic field, *B*_z_ forces previously vitrified nanoparticle inclusions (${d}_{{\rm{v}}}$ > $\bar{d}$) to align along the magnetic flux within the polymer droplet. **b** Optical micrographs of a unidirectionally magnetized particle and a control particle prepared without a magnetic field. **c** Rotating motion of the single magnetized particle using an external magnetic field, *B*_z_. **d** Collective actuated behavior of a group of the magnetized particles.

Unidirectionally magnetized particles are made by applying an external magnetic field, *B*_z_ to uncured composite droplets with ferromagnetic Fe inclusions, as shown in Fig. 5. The magnetic field, *B*_z_ aligns the internal inclusions along the field, otherwise a high viscosity of the polymer resin upholds $\bar{d}$ < ${d}_{{\rm{v}}}$ and allows the inclusions to remain randomly distributed (Fig. 5a). Figure 5b shows the imposed order inside a unidirectionally magnetized particle and a control particle with a random inclusion distribution when no magnetic field is applied during solidification. The programmed magnetism of each particle enables external control over single particle motions (Fig. 5c and Supplementary Movie 5), and furthermore collective particle behaviors (Fig. 5d). Likewise, the anisotropy of these mass-produced particles allows for controllable robotic motions, which are not achievable using isotropic particles. We anticipate our static-state particle fabrication process to shift paradigms in designing and manufacturing intelligent particles that have been mostly relied on microfluidic processes of low process yields.

In sum, we present a method to manufacture particles using the rapid vitrification of a thixotropic medium. The vitrification of the shear-thinning medium allows the distributed droplets to solidify, devoid of shearing forces typically required during the emulsion process. This lack of applied shear results in a significant improvement in the consistency of particle shape and size within a given batch, with a notable process yield rate higher than 95%. Furthermore, we used the static-state process to mass-produce anisotropic Janus particles and unidirectionally magnetized particles. The presented method is a technical breakthrough for future academic research into particulate composites, dispersion inks, and granular metamaterials, due to its simple implementation and high adaptability to many existing intelligent particle designs. Moreover, the process is eco-friendly (no synthetic materials used) and energy-efficient (no extra power consumption from redundant stirring), which will streamline the technology transfer of this static-state particle fabrication to industry-scale manufacturing.

## Methods

### Materials

Corn starch granules (MFG 2006779) were purchased from Argo (Oakbrook, IL), and used to prepare starch/water mediums by mixing with DI-water at various concentrations of 5–25 wt% and gelatinizing the granules at 95 °C for 1 h. Amylase enzyme (SKU: 5BHBZZ7100B) was purchased from BSG (Shakopee, MN). Field’s Metal blocks (SKU: LMP144) were purchased from RotoMetals (San Leandro, CA) and used as-is. Two-part silicone resin EcoFlex 00-30 and one-part RTV silicone resin Semicosil 964 were purchased from Smooth-On (Macungie, PA) and Wacker (Adrian, MI), respectively. Iron powder (<10 μm) (SKU: 267953) and silver-coated ferrite particles (~25 μm) (SM25P20) were purchased from Sigma Aldrich (St. Louis, MO) and Potters Industries, LLC respectively, and used as fillers of anisotropic particles. Hydrogen peroxide and Triton X-100 (SKU: X100) were purchased from Sigma Aldrich (St. Louis, MO) and used as reaction medium and surfactant for propelling Janus particles.

### Preparation of Field’s Metal particles

A bulk of 10 g Field’s Metal was added to the gelatinized starch/DI-water medium of 200 ml and melted for 2 min at 95 °C. A shear mixer broke up the molten Field’s Metal at various shear rates and removed off after 10 min. Two types of shear mixer were used to emulsify the molten Field’s Metal: a 10-speed overhead mixer (RW16, IKA) with a 3-bladed stirrer of 56 mm in diameter (Item# UX-50703-31, IKA), and a 6-speed lab homogenizer (LabGEN 850, Cole-Parmer) with a 20 mm saw-tooth generator (Item# UX-04727-41, Cole-Parmer). Applied shear rates are ranging from 600 rpm to 9,500 rpm when using the overhead mixer, and from 17,500 rpm to 24,000 rpm when using the homogenizer. The entire medium containing micro-droplets of the molten Field’s Metal was cooled down at room temperature to solidify the molten metal.

An aqueous solution of 5 wt% amylase enzyme of 100 ml was then added to the resulting batch and the temperature was raised to 40 °C to catalyze hydrolysis of the contained gelatinized starch. The hydrolysis reaction was performed for 1–2 h under a gentle shearing (i.e., a magnetic stir bar or a tube rotator) to ensure the contact between the enzyme and the starch. Depolymerization of the starch from the hydrolysis caused Field’s Metal particles to rapidly sediment and the particles were easily retrieved. Such a hydrolysis step was especially important for fabricating smaller/polymeric particles because long-chains of the pristine starch remain adhered to the particles until being cut into the short-chain glucose. Retrieved particles were additionally washed using DI-water for 3 times and dried at room condition.

### Experimental methods

Rheological behavior of thixotropic gelatinized starch/DI-water medium was measured using RSA-G2 from TA Instruments with a parallel plates fixture of 15 mm in diameter. Frequency sweep was performed at 80 °C with an oscillatory strain of 1.0%, and a solvent trap was used to prevent the premature evaporation of DI-water during the measurements. Stress relaxation was performed at room temperature with the initial strain of 1.0% and ${\sigma }_{{\rm{v}}}$ is predicted from a trendline, at *t* = 1 h, achieved of the logarithmic experimental data. The surface tension of polymer resins in water was measured via a pendant drop method at room temperature. A drop of the resin formed at an ID 0.34 mm syringe nozzle (23 G, BSTEAN) was imaged using a Grasshopper camera (Part# GS3-U3-51S5M-C, FLIR) and an imaging software ImageJ analyzed the radii of the drop. The same method was applied, but the water at 70 °C, to measure the surface tension of the molten Field’s Metal. Optical micrographs of particles were obtained by Smartzoom 5 from Zeiss and scanning electron micrographs (SEM images) were obtained using SU-70 from Hitachi where energy-dispersive X-ray spectroscopy (EDS) was also performed at 10.0 kV. XRD data were obtained using Rigaku SmartLab X-ray diffractometer equipped with Cu Kα radiation under Bragg-Brentano focusing mode at step 0.02° over a 2*θ* range of 10° to 100°. The phase identification of the particles was analyzed using PDXL software.

The particle size distribution was achieved by two different methods: sieve analysis and imaging analysis. Sieve analysis was performed by sieving a batch of fabricated particles using a set of sieves and constructing a relevant cumulative distribution curve. The mesh sizes of used sieves were 850, 355, 212, 106, 75, 45 μm purchased from DUAL MFG (Franklin Park, IL) and the cumulative distribution curve was predicted referencing the Weibull cumulative probability function as,

3 $$F(x)=1-{e}^{-{(\frac{x}{\lambda })}^{k}}$$

where *k* is the Weibull shape factor and *λ* is the scale factor.

Imaging analysis for the size distribution was performed on the particles collected on each sieve, respectively. Backlight optical micrographs of the collected particles were taken, and the Image J software was used to calculate the frequency of particle sizes pertinent to each sieve. The overall particle distribution *Q*(*x*_*k*_) was obtained by multiplying this particle size frequency (from the imaging analysis) with the weight fraction (from the sieve analysis) as,

4 $$Q({x}_{k})={w}_{i}\times \frac{{p}_{k}{x}_{k}^{3}}{\mathop{\sum }\limits_{j=1}^{n}{p}_{j}{x}_{j}^{3}}\,(i=1,2,\ldots ,6\,{\rm{and}}\,1\le k\le n)$$

where *w*_*i*_ is the weight fraction of particles collected on a sieve *i* (total 6 sieves), *p*_*j*_ is the frequency of particle at size *x*_*j*_ in the sieve *i*. As a result, the mean particle diameter $\overline{d}$ and normalized standard deviation $\bar{s}$ were achieved as,

5 $$\bar{d}=\mathop{\sum }\limits_{k=1}^{N}{x}_{k}Q({x}_{k})$$

6 $$\bar{s}=\frac{s}{\bar{d}}=\frac{1}{\bar{d}}\sqrt{\mathop{\sum }\limits_{k=1}^{N}{x}_{k}^{2}Q({x}_{k})-{\left(\mathop{\sum }\limits_{k=1}^{N}{x}_{k}Q({x}_{k})\right)}^{2}}$$

where *N* is the total number of particles.

### Simulation of particle collisions

2D simulation of particle collisions lacking a starch inter-particle layer was performed by assuming the particles moving in the medium as deformable sticky particles. Deformable particles were modeled using polygons in which the vertices were freely jointed, but the area was conserved. Stickiness, that gives rise to particle coalescence after their collision, was included in the simulation via an energy term proportional to the particle surface.

The total number of particle collisions with respect to viscosities of the medium was obtained in a 3D setting by assuming no collisions to cause particle coalescence. Therefore, the total number of particles in a controlled 3D space was maintained the same over time. See Supplementary Note 2 for details.

### Preparation of anisotropic particles

Self-propelling Janus particles were fabricated by emulsifying a mixture of 1 ml semicosil-964, 0.3 ml toluene (solvent) and 0.5 g silver-coated ferrite particles in 50 ml gelatinized starch/water medium (10 wt% of starch concentration). The emulsification was performed at room temperature for 30 s using the homogenizer applying a shearing rate of 600 rpm. The resulting vitrified emulsion then underwent a resting period of 3 h to allow the silver-coated ferrite particles to sediment and the polymer resin to cure.

Unidirectionally magnetized stretchable particles were fabricated by emulsifying a mixture of 2 ml Eco-Flex 00-30 (two parts mixed at 1:1 by weight) and 0.8 g iron powder in 50 ml gelatinized starch/water medium (10 wt% of starch concentration). The emulsification was performed at room temperature for 1 min using the overhead mixer that applies a shearing rate of 600 rpm. The resulting vitrified emulsion was placed on top of a 50 mm neodymium magnet (5862K43, McMaster) to unidirectionally align the iron powders and remained for 3 h to allow the polymer resin to cure. Both Janus particles and unidirectionally magnetized particles were cleaned via the aforementioned procedure that uses amylase enzyme.

### Demonstration of robotic anisotropic particles

Self-propelling particles were realized by placing Janus particles in a 100 mm PYREX petri dish, and 30 wt% hydrogen peroxide solution was poured to initiate a reaction that creates oxygen bubbles on the side of the Janus particle surfaces covered with silver-coated ferrite particles. A surfactant Triton X-100 (0.5 wt%) was added to the solution to spur the oxygen bubbles on to be detached from the particle surface. Since the heavier ferrite particles settle and become deposited from the very bottom of polymer droplets, we hypothesize the amount of the ferrite particles required to impart the self-propelling ability will be notably less than what is used in this study (0.5 g per 1 ml resin). The magnetized particles were actuated by placing into a rotating magnetic field.

## Supplementary information

Supplementary information

Peer Review File

Description of Additional Supplementary Files

Supplementary Movie 1

Supplementary Movie 2

Supplementary Movie 3

Supplementary Movie 4

Supplementary Movie 5

## Data Availability

The data that support the findings of this study are available from the corresponding authors upon reasonable request.
