# Supplementary material for: Static-state particle fabrication via rapid vitrification of a thixotropic medium
Source: Nat Commun. 2021 Jun 18;12:3768. doi: 10.1038/s41467-021-23992-2 (PMC8213858; doi:10.1038/s41467-021-23992-2)
Supplement: Supplementary file 1 — Supplementary information [file 41467_2021_23992_MOESM1_ESM.pdf]

Supplementary Information

# Static-State Particle Fabrication via Rapid Vitrification of a Thixotropic Medium

Sang Yup Kim[1,2], Shanliangzi Liu[1,3], Sungwoo Sohn[1], Jane Jacobs[1], Mark D. Shattuck[4], Corey S. O'Hern[1], Jan Schroers[1], Michael Loewenberg[5], and Rebecca Kramer-Bottiglio[1*]

[1]Department of Mechanical Engineering and Materials Science, Yale University, 9 Hillhouse Ave, New Haven, CT 06520, USA

[2]Department of Mechanical Engineering, Sogang University, 35 Baekbeom-ro, Seoul, Republic of Korea

[3]School of Mechanical Engineering, Purdue University, 585 Purdue Mall, West Lafayette, IN 47907, USA

[4]Department of Physics, City University of New York, 42 Street, New York, NY 10017, USA

[5]Department of Chemical and Environmental Engineering, Yale University, 9 Hillhouse Ave, New Haven, CT 06520, USA

[*]Correspondence to: rebecca.kramer@yale.edu

## SUPPLEMENTARY FIGURES

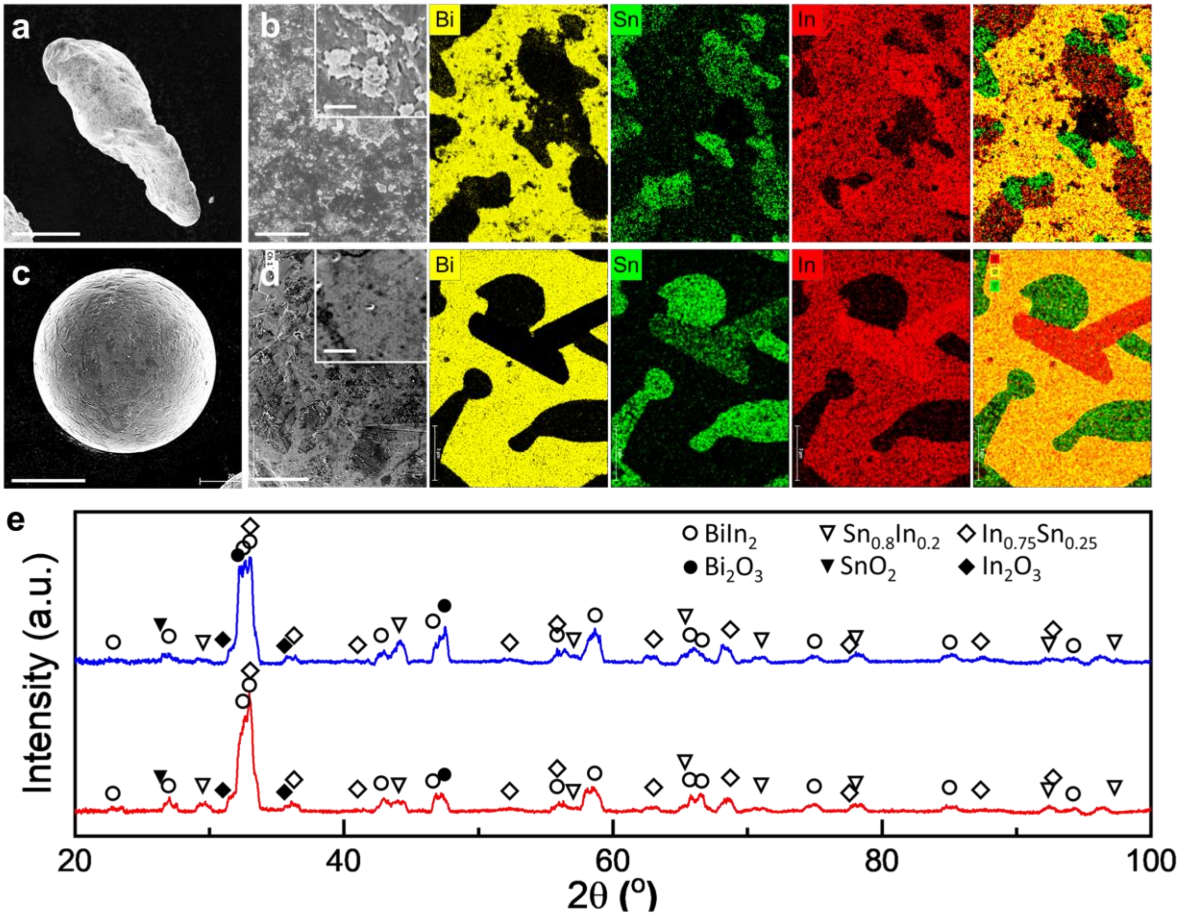


Supplementary Fig. 1 | **Microstructure of Field's Metal (FM) particles fabricated under different environments.** **a-d** SEM images and EDS results of FM particles fabricated at dynamic-state in water medium (**a** and **b**) and static-state in starch/water medium (**c** and **d**). EDS results of Bi (yellow), Sn (green), In (red), and their composite images are corresponded with SEM images **b** and **d**, respectively. **e** XRD results of each FM particle (blue: dynamic-state, red: static-state). See Supplementary Note 1 for details. Scale bars: (**a**) 50 μm, (**c**) 20 μm, (**b** and **d**) 3 μm, and (inset in **b** and **d**) 500 nm.

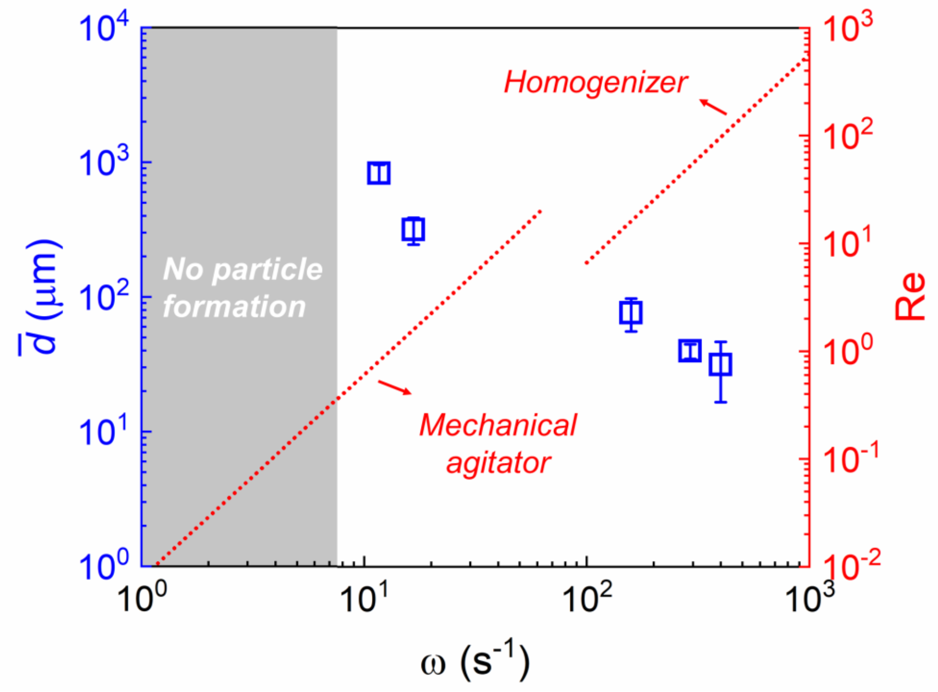


Supplementary Fig. 2 | **Diameter of Field's Metal particles as a function of shear rate.** Shear rate $\omega$ controls the mean particle diameter $\bar{d}$. Mechanical agitator and homogenizer are used for emulsification at low and high $\omega$, respectively, and each Reynold's number Re is calculated. Error bars denote one standard deviation.

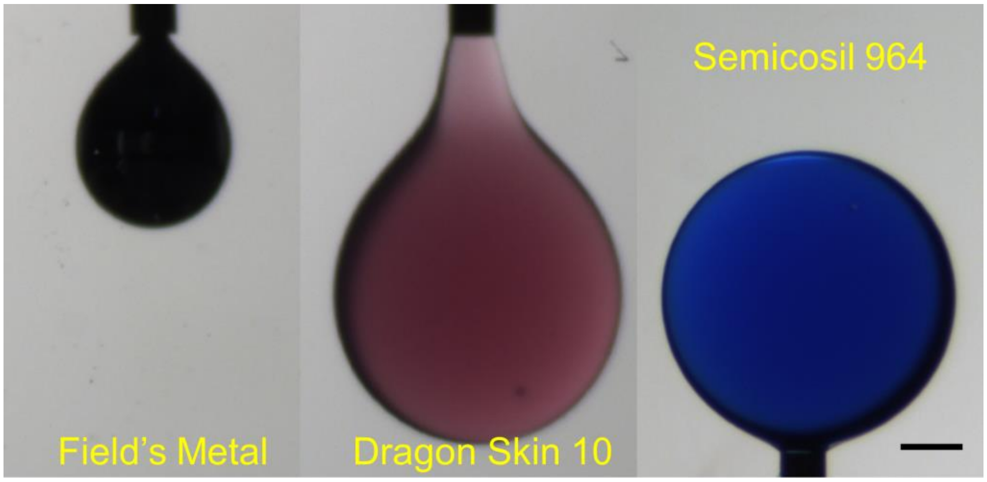


Supplementary Fig. 3 | **Pendant drop measurement of distributed materials.** Viscosities $\eta_C$, densities $\rho_d$, and surface tensions $\gamma$ of each material are Field's Metal ($\eta_C$ = 0.027 Pa·s, $\rho_d$ = 7,880 kg/m$^3$, $\gamma$ = 302.4 mN/m), Dragon Skin 10 ($\eta_C$ = 23 Pa·s, $\rho_d$ = 1,070 kg/m$^3$, $\gamma$ = 12.6 mN/m), and Semicosil 964 ($\eta_C$ = 0.7 Pa·s, $\rho_d$ = 990 kg/m$^3$, $\gamma$ = 5.8 mN/m). Scale bar: 2 mm.

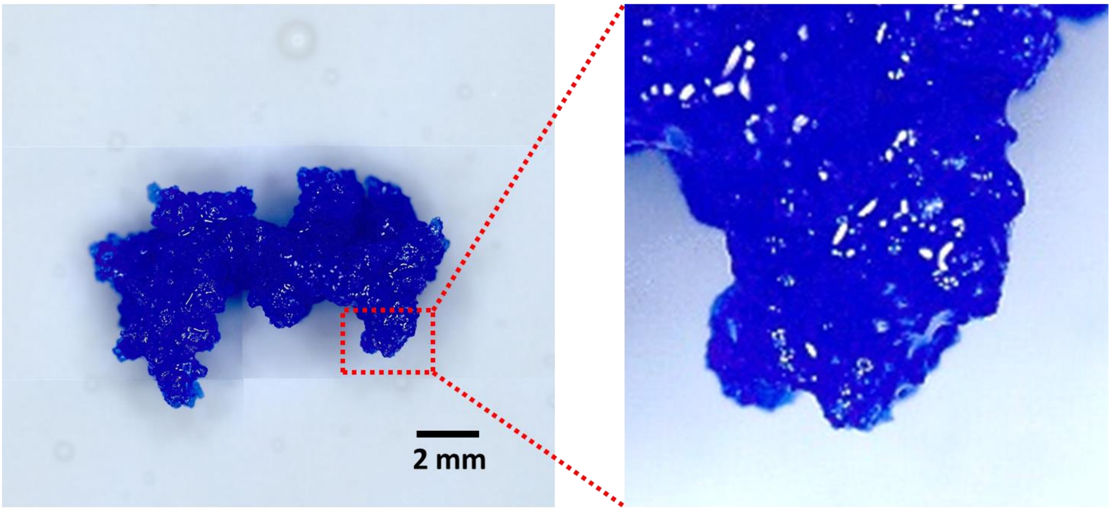


Supplementary Fig. 4 | **Optical micrograph of agglomerated Semicosil 964 (SC) particles.** SC particles fabricated at $\phi$ = 2.5 wt.% turn into an agglomerated piece due to particle coalescence after the collision.

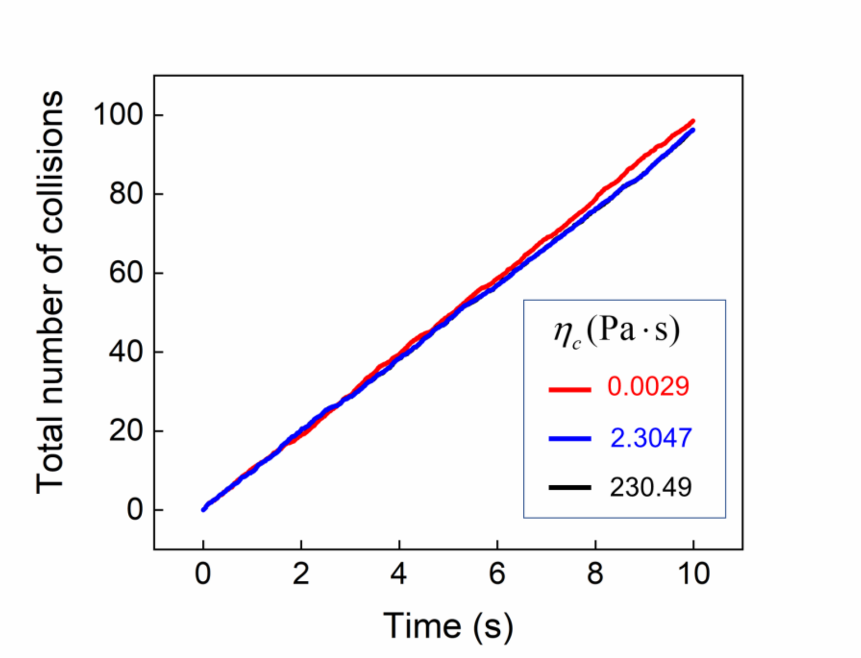


Supplementary Fig. 5 | **Simulation of particle collisions.** The number of collisions of SC particles is linearly increasing with time, and the viscosity of the continuous phase $\eta_C$ has an insignificant effect on the collision rate.

## SUPPLEMENTARY NOTES

Supplementary Note 1 | **Microstructure of FM particles**

Here we detail structural and microstructural characteristics of Field's Metal particles fabricated under both static-state and dynamic-state conditions. A particle fabricated under dynamic-state conditions in a water medium exhibits an irregular shape with a rough surface (Supplementary Fig. 1a), while a particle fabricated under static-state conditions exhibits a spherical shape with a smooth surface (Supplementary Fig. 1c). We believe that the difference in shape and surface texture originates from particle collisions during solidification and corresponding phase segregation[1]. Supplementary Fig. 1b shows surface features ranging from 50 to 500 nm covering the particle fabricated under dynamic-state conditions, which may be due to: a) multiscale particle collisions, b) cracked/shattered surface oxides during particle collisions, and/or c) uneven integration of materials while particles are merging. X-ray diffraction (XRD) analysis reveals pronounced $Bi_2O_3$ peaks in particles fabricated through the dynamic-state condition (marked by solid circles in Supplementary Fig. 1e), which supports the hypothesis that oxide fragments contribute to the surface features.

Oxide fragments are also seen in the mixed EDS elemental mapping (far right in Supplementary Fig. 1b) as black dots. We note that the tendency of oxidation quantified by Gibbs free energy of oxidation follows the order of $In_2O_3 > SnO_2 > Bi_2O_3$ [2]. Thus, $Bi_2O_3$ is not expected to form on the surface of Bi-Sn-In alloys as a significant oxide[3]. This observed deviation from the expected thermodynamic condition indicates an opportunity to form unusual oxide products using the dynamic-state process. In contrast, the XRD pattern from particles fabricated under static-state conditions shows a lower quantity of $Bi_2O_3$, which is in agreement with the expected oxidation of this material system[2].

Micrographs with EDS elemental mappings from both particles (in Supplementary Fig. 1b and d) reveal typical ternary eutectic microstructures. Based on the curved boundaries between phases, we describe these microstructures as non-faceted / non-faceted type eutectic in a smaller category. The combined results from the XRD and EDS mappings suggest that both ternary eutectics consist of $BiIn_2$, $Sn_{0.8}In_{0.2}$ and $In_{0.75}Sn_{0.25}$ phases, which is in agreement with other studies[4,5].

Supplementary Note 2 | **Simulation of particle collisions**

To visualize the coalescence of particles following the collision, we simulate 2D deformable sticky particles. Deformable particles are modeled using polygons in which the vertices are freely jointed, but the area(mass) is conserved[6,7]. To add stickiness, we include an energy term proportional to the perimeter (surface). This term is the 2D equivalent of surface tension. The surface energy between particles and fluid is 10 times that of the energy between two particles. The deformable sticky particles are placed in a periodic Lees-Edwards[8] shear flow. Because of the shear stress on the particles in the flow, they are deformed into an elliptical shape (see t = 3sec). When particles collide, they may stick and coalesce due to the lower energy of the particle-particle interface. The shear flow also competes with the stickiness to elongate and potentially break the clusters up.

To calculate an effect of the medium's viscosity on the collision frequency of moving particles, We simulate 180 spheres of diameter 1 μm × (1 + 0.25 × N(0,1)), where N(0,1) is zero mean standard deviation of one random number. The particles are placed at random in a small parallelepiped $L_x \times L_y \times L_z$ of 62.8 μm × 61.9 μm × 10.0 μm. The $L_z$ direction is fixed to 10 times the mean particle diameter, $L_x$ is determined by requiring a fixed fraction (1 / 300) of the

experimental domain, and $L_y$ is set so that the 180 spheres give a number density of $5 \times 10^6$ particles/ml. Gravity points downward in the $L_y$ direction. We use Lees-Edwards shear boundary conditions[8] to create a simple shear in the $L_y$ direction with shear rate $\omega = 16$. The shear flow is specified for the fluid and the particles follow by a Stokian drag force:

$$\mathbf{F}_{\text{drag}} = -B(\mathbf{v}_{\text{fluid}}(y) - \mathbf{v}_{\text{particle}})$$

where $B = 6\pi\eta R M^{-1}$, $\eta$ is the viscosity of the fluid, $R$ and $M$ are the radius and mass of the particle, $\mathbf{v}(y) = \omega\hat{x}$. In all cases, the particles reach terminal velocity in a few time steps and follow the falling shear flow velocity. We then record the number of collisions in the small domain over time. This measurement is averaged over 100 realizations. The number of collisions can be converted to collision per ml by dividing by the volume $L_x \times L_y \times L_z$.

## SUPPLEMENTARY DISCUSSION

Supplementary Discussion 1 | **Relaxation time of the droplet interface in a vitrified medium**

The vitrification time—time required for a medium to recover its zero-shear value after shearing stops—and the relaxation time of droplet interface are additional factors that can determine the shape of droplets. For example, if a medium reaches the vitrification earlier than the relaxation of distributed droplets' interface (vitrification time < relaxation time), the droplets could gain irregular shapes.

All the particles which we fabricated in this study were nearly perfect spheres, indicating that the surface tension of the droplet interface was the dominant force to determine the shape of the droplets. This particle morphology indicates that the droplets had sufficient time to relax in the medium until their solidification (vitrification time > relaxation time), presumably because of the stark difference in length scales of the medium (approx. $10^2$ ml) and the droplets (approx. $10^{-5}$ ml).

Supplementary Discussion 2 | **Processable maximum volume fraction of the distributed phase**

The maximum volume fraction of the distributed phase processable by an emulsion process is dependent on the characteristics of phase inversion[9,10], and physical properties (e.g. viscosity, surface tension, density) of both the distributed and continuous phase. While we did not explicitly characterize this value for each of the material systems we present, we hypothesize the maximum volume fraction of the target material in a vitrifying medium would be similar to that in a non-vitrifying medium, if the mediums have similar viscosities prior to vitrification (during shear). However, vitrification of the medium will yield more monodisperse particles by preventing collisions and coalescence during particle solidification.

## SUPPLEMETARY REFERENCES

1. Çınar, S., Tevis, I. D., Chen, J. & Thuo, M. Mechanical fracturing of core-shell undercooled metal particles for heat-free soldering. *Sci. Rep.* **6**, 21864 (2016).
2. Zavabeti, A. *et al.* A liquid metal reaction environment for the room-temperature synthesis of atomically thin metal oxides. *Science (80-. ).* **358**, 332 LP – 335 (2017).
3. Idrus-Saidi, S. A. *et al.* Liquid metal core–shell structures functionalised via mechanical agitation: the example of Field's metal. *J. Mater. Chem. A* **7**, 17876–17887 (2019).
4. Ruggiero, M. A. & Rutter, J. W. Origin of microstructure in the 332 K eutectic of the Bi-In-Sn system. *Mater. Sci. Technol.* **13**, 5–11 (1997).
5. Çadırlı, E. *et al.* The effect of growth rate on microstructure and microindentation hardness in the In–Bi–Sn ternary alloy at low melting point. *J. Alloys Compd.* **470**, 150–156 (2009).
6. Boromand, A., Signoriello, A., Ye, F., O'Hern, C. S. & Shattuck, M. D. Jamming of deformable polygons. *Phys. Rev. Lett.* **121**, 248003 (2018).
7. Boromand, A. *et al.* The role of deformability in determining the structural and mechanical properties of bubbles and emulsions. *Soft Matter* **15**, 5854–5865 (2019).
8. Wagner, A. J. & Pagonabarraga, I. Lees–Edwards boundary conditions for lattice Boltzmann. *J. Stat. Phys.* **107**, 521–537 (2002).
9. Kumar, A. *et al.* Recent developments in phase inversion emulsification. *Industrial & Engineering Chemistry Research* **54**, 8375-8396 (2015).
10. Bremond, N. *et al.* Propagation of drop coalescence in a two-dimensional emulsion: A route towards phase inversion. *Phys. Rev. Lett.* **106**, 214502 (2011).
