# Supplementary material for: Static-state particle fabrication via rapid vitrification of a thixotropic medium
Source: Nat Commun. 2021 Jun 18;12:3768. doi: 10.1038/s41467-021-23992-2 (PMC8213858; doi:10.1038/s41467-021-23992-2)
Supplement: Supplementary file 3 — Description of Additional Supplementary Files [file 41467_2021_23992_MOESM3_ESM.pdf]

Description of Additional Supplementary Files

File name: Supplementary Movie 1
Description: Static-state fabrication of FM particles

File name: Supplementary Movie 2
Description: Simulation of particle collisions

File name: Supplementary Movie 3
Description: Fabrication of Janus particles

File name: Supplementary Movie 4
Description: Self-propelling Janus particle

File name: Supplementary Movie 5
Description: Control of unidirectionally magnetized particles
