# Supplementary material for: Static-state particle fabrication via rapid vitrification of a thixotropic medium
Source: Nat Commun. 2021 Jun 18;12:3768. doi: 10.1038/s41467-021-23992-2 (PMC8213858; doi:10.1038/s41467-021-23992-2)
Supplement: Supplementary file 2 — Peer Review File [file 41467_2021_23992_MOESM2_ESM.pdf]

Editorial Note: This manuscript has been previously reviewed at another journal that is not operating a transparent peer review scheme.
